# iJGVD: an integrative Japanese genome variation database based on whole-genome sequencing

**DOI:** 10.1038/hgv.2015.50

**Published:** 2015-11-26

**Authors:** Yumi Yamaguchi-Kabata, Naoki Nariai, Yosuke Kawai, Yukuto Sato, Kaname Kojima, Minoru Tateno, Fumiki Katsuoka, Jun Yasuda, Masayuki Yamamoto, Masao Nagasaki

**Affiliations:** 1 Department of Integrative Genomics, Tohoku Medical Megabank Organization, Tohoku University, Sendai, Japan; 2 Graduate School of Medicine, Tohoku University, Sendai, Japan; 3 Department of Cohort Genome Information Analysis, Tohoku Medical Megabank Organization, Tohoku University, Sendai, Japan; 4 Graduate School of Information Sciences, Tohoku University, Sendai, Japan

## Abstract

The integrative Japanese Genome Variation Database (iJGVD; http://ijgvd.megabank.tohoku.ac.jp/) provides genomic variation data detected by whole-genome sequencing (WGS) of Japanese individuals. Specifically, the database contains variants detected by WGS of 1,070 individuals who participated in a genome cohort study of the Tohoku Medical Megabank Project. In the first release, iJGVD includes >4,300,000 autosomal single nucleotide variants (SNVs) whose minor allele frequencies are >5.0%.

Since the completion of the Human Genome Project,^1^ many studies have focused on the detection and characterization of genomic variants.^2–4^ In Japan, a gene-based single nucleotide polymorphism (SNP) discovery project as part of the Japanese Millennium Genome Project reported >190,000 variants and catalogued the SNPs in the JSNP database.^5,6^ This catalogue of high-quality SNPs was the foundation that led to the early success of genome-wide association studies in Japan.^7^ The International HapMap Project^8–10^ has produced genome-wide SNP genotype data for major ethnic groups including the Japanese population, and these data have facilitated genome-wide association studies.

Although reports of common variants and their frequencies are accumulating for various populations, it is difficult to avoid ascertainment biases (e.g., well-known SNPs or tag SNPs are disproportionately examined). Many low-frequency variants remain undetected or have unknown frequencies. A catalogue of genomic variants from WGS and estimates of variant frequencies for each population are needed to provide a foundation for genomic medicine. The 1000 Genomes Project (1KGP)^11^ involved low-coverage WGS and high-coverage exome sequencing for >1,000 individuals, including 89 Japanese samples, and the data is widely used for genotype imputation. However, the sample sizes for individual populations are insufficient to obtain reliable allele frequencies. Therefore, high-coverage WGS of a larger number of individuals for a target population is desired to construct a variant catalogue with reliable allele frequencies, including rare variants.

To make a reference panel of genomic variation for the Japanese population, we sequenced whole genomes of 1,070 cohort participants, and detected genomic variants including SNVs, indels and structural variants.^12^ This variant set formed a reference panel for the Japanese population, which we refer to as ‘1KJPN.’ We released the comprehensive catalogue of SNV frequencies for alleles whose frequencies are >5% among the 1,070 individuals. The current release of iJGVD provides allele frequency data for 4,301,546 autosomal SNVs.

The set of variants in iJGVD was released from 1KJPN, which was constructed with data from the WGS of 1,070 healthy Japanese individuals in the Tohoku Medical Megabank Project.^12^ The 1KJPN subjects were adult individuals (age ⩾20 years) whose Japanese ancestry was confirmed, and close-relatives were excluded (see Supplementary Figure 1 for statistics regarding age and sex). All participants gave written informed consent.

In this project, the genomic DNA of 1,070 subjects obtained from peripheral blood samples was subjected to paired-end sequencing using the Illumina HiSeq 2500 platform. All sequencing libraries were constructed based on PCR-free methods.^13^ The sequence reads were mapped onto the human reference genome, assembly GRCh37/hg19, with decoy sequences (hs37d5) and an average sequencing coverage of 32.4× for full-length autosomal chromosomes. Variant calling and subsequent filtering were performed by an in-house bioinformatics pipeline.^14,15^ The details of methods and quality controls are described in Nagasaki *et al.*^12^

Among the total variants in 1KJPN, autosomal SNVs whose minor allele frequencies were >5% were selected. These SNVs were annotated with their corresponding database SNP (dbSNP) IDs and their effects on gene products were predicted using SnpEff^16^. SNVs were selected if the variants were reported in dbSNP138^3^, and the iJGVD release (Version 1.0) included a final sample size of 4,301,546 SNVs.

The iJGVD system consists of (i) the relational database and (ii) the web server (Figure 1a). The relational database (using MySQL 5.1.73) for iJGVD includes SNV alleles, genomic positions based on the GRCh37/hg19 coordinates, allele frequencies, the corresponding dbSNP IDs, *P* values for the Hardy–Weinberg equilibrium test, gene annotations and so on. The web server consists of functions to search SNVs and explore the region surrounding an SNV based on chromosome coordinates. The web server and exploration functions were implemented in PHP 5.3.3 and JBrowse 1.11.5, respectively.

Among the 4,301,546 SNVs, 1.72% were located in exonic regions (i.e., untranslated regions or coding regions). The minor allele frequency distribution for the SNVs in iJGVD was examined (Table 1). The SNV counts for each frequency class were not uniform, and the sample was enriched for low-frequency SNVs.

We compared the allele frequencies of SNVs in iJGVD with those of SNVs in HapMap3^10^ JPT (Japanese from Tokyo) individuals (Figure 2a). The allele frequencies in the two populations were very similar (the correlation coefficient was 0.99). We also tested statistical difference in allele counts between ToMMo 1KJPN and HapMap3 JPT, and found that only a small fraction (0.022%, 226 out of 1,020,909) of SNVs showed *P* values of <10^−8^ (see Supplementary Figure 2 for QQ-plots). This fraction of SNVs with small *P* values was very similar with that for the comparison between NGS data and SNP array data in the JPT population (Figure 2b).

SNVs in iJGVD can be searched by specifying the gene symbol, rsSNP ID, or genomic position (Figures 1b and c). Hits are displayed in a table of SNVs with allele frequencies in sequential order based on their genomic coordinates. The table can be downloaded as a text file by clicking ‘Download Table.’ SNVs can also be queried using the genome browser by specifying the chromosome and genomic position. The genome browser (Figure 1d) provides graphical views of the genomic location of SNVs with locations of known genes and other SNVs in dbSNP.

We constructed a public database of genomic variants with allele frequencies for the Japanese population. Variant databases for the Japanese population to date have been based on targeted SNP typing^6^ or whole-exome sequencing.^17^ iJGVD is the first database of genomic variants for Japanese individuals based on high-coverage WGS. A set of variants and the corresponding frequency information from WGS would provide a comprehensive platform for finding disease-causing variants because they can be found in non-coding regions. The allele frequencies of SNVs in iJGVD and in the HapMap3 JPT population are highly correlated (Figure 2b). Furthermore, our database contains allele frequencies for more than three million additional high-quality SNVs that were not genotyped in the HapMap3 project. We recently designed a genotyping chip, ‘Japonica Array’, which was optimized for the Japanese population,^18^ and probes for autosomal SNPs on Japonica Array can be seen in iJGVD.

We plan to improve the usefulness of iJGVD by adding biological annotations for SNVs and expanding search options using these annotations. Furthermore, information of linkage disequilibrium will be considered for additional data. Although iJGVD contains only SNV information at present, insertions, deletions and other structural variants will be included after quality control processes are implemented. We believe that our open variant data will be useful in medical genomics, especially for comparisons of allele frequencies in iJGVD with those of the patient group for a target disease to identify disease-causing variants.

All SNV frequency data in iJGVD are available from the National Bioscience Database Center Human Database (http://humandbs.biosciencedbc.jp/) under accession hum0015.

## Figures and Tables

**Figure 1:**
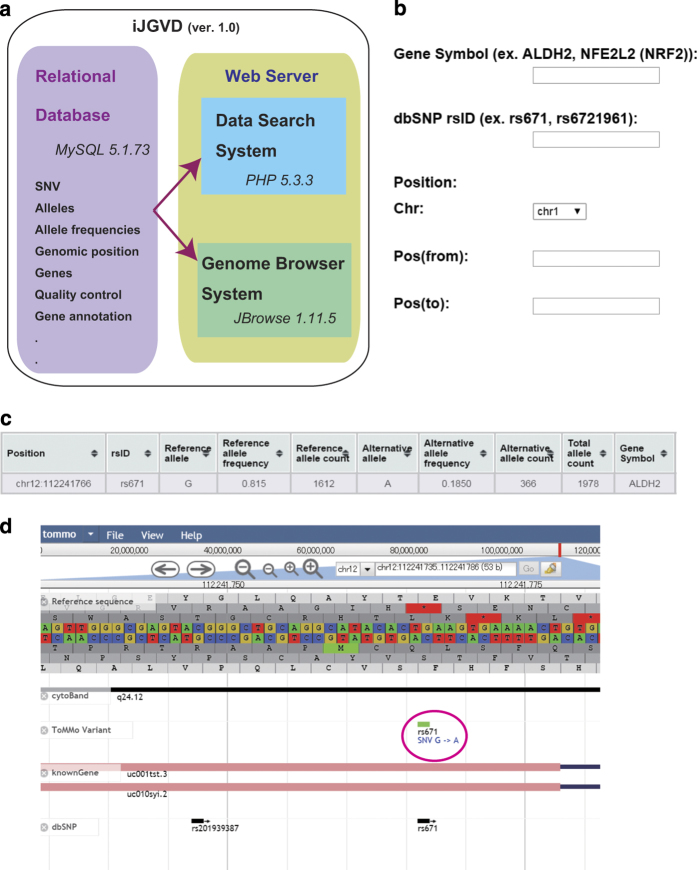
Schema of the systems and graphical user interfaces of iJGVD. (**a**) Schematic diagram of the iJGVD systems. (**b**–**d**) Graphical user interfaces for iJGVD. (**b**) SNV searches are initiated at the top page by specifying a gene, dbSNP ID, or genomic region. (**c**) SNV allele frequencies are displayed in a table, and rs671 is shown as an example. (**d**) A graphical view of the SNV location in the genome browser. iJGVD, integrative Japanese Genome Variation Database ; dbSNP, database single nucleotide polymorphism; SNV, single nucleotide variant.

**Figure 2:**
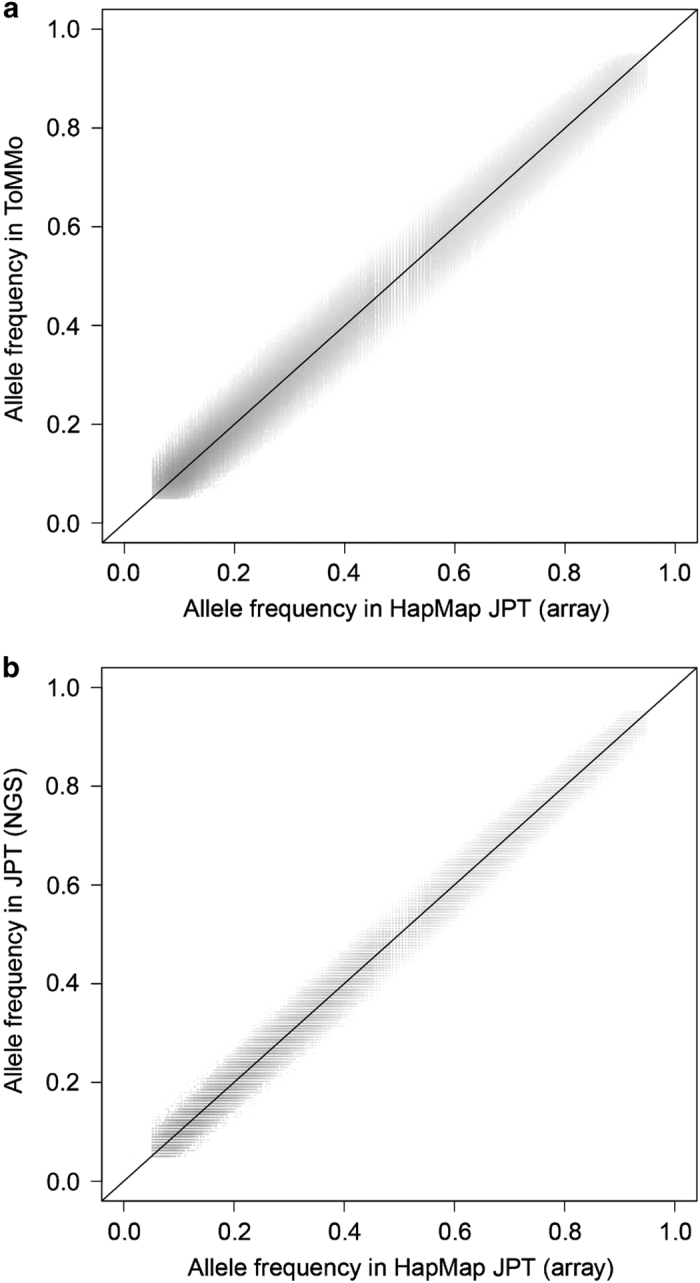
Comparison of SNV allele frequencies in ToMMo 1KJPN with those of HapMap JPT. (**a**) Non-reference SNV allele frequencies in ToMMo 1KJPN (*y* axis) are shown with those in HapMap3 JPT individuals (*n*=86; *x* axis) for 1,020,909 overlapping SNVs in a two-dimensional scatter plot. (**b**) Non-reference SNV allele frequencies in 1KGP JPT individuals (*n*=89) by whole-genome sequencing (*y* axis) are plotted against those in HapMap3 JPT individuals (*n*=86; *x* axis) for 1,061,165 autosomal SNVs. JPT, Japanese from Tokyo; SNV, single nucleotide variant.

**Table 1:** Number of SNVs in iJGVD by frequency class and functional category

*Functional category*	*Frequency class*								
	0.05–0.10	0.10–0.15	0.15–0.20	0.20–0.25	0.25–0.30	0.30–0.35	0.35–0.40	0.40–0.45	0.45–0.50
Nonsynonymous	3,114	2,113	1,726	1,393	1,248	1,181	1,170	1,089	995
Synonymous	3,228	2,169	1,817	1,565	1,450	1,458	1,333	1,266	1,268
5′ UTR	1,980	1,310	1,208	939	866	849	856	831	745
3′ UTR	7,215	4,958	4,135	3,555	3,128	3,185	2,923	2,948	2,906
Splice donor site	25	10	6	4	5	9	7	8	6
Splice acceptor site	8	11	7	11	3	5	5	6	8
Intron	307,422	219,990	187,246	163,319	152,763	143,780	136,719	131,543	129,083
Others	499,044	366,535	313,854	283,193	255,771	245,457	234,201	229,951	225,074
Total	822,036	597,096	509,999	453,979	415,234	395,924	377,214	367,642	360,085

Abbreviations: iJGVD, integrative Japanese Genome Variation Database ; SNVs, single nucleotide variants; UTR, untranslated region.
