# Supplementary material for: iJGVD: an integrative Japanese genome variation database based on whole-genome sequencing
Source: Hum Genome Var. 2015 Nov 26;2:15050–. doi: 10.1038/hgv.2015.50 (PMC4785574; doi:10.1038/hgv.2015.50)

**Supplementary Figure 1**

**Properties of 1,070 Japanese subjects.**

The numbers of subjects for each age group are shown for males and females. The subjects were grouped according to their age at the end of year 2015.


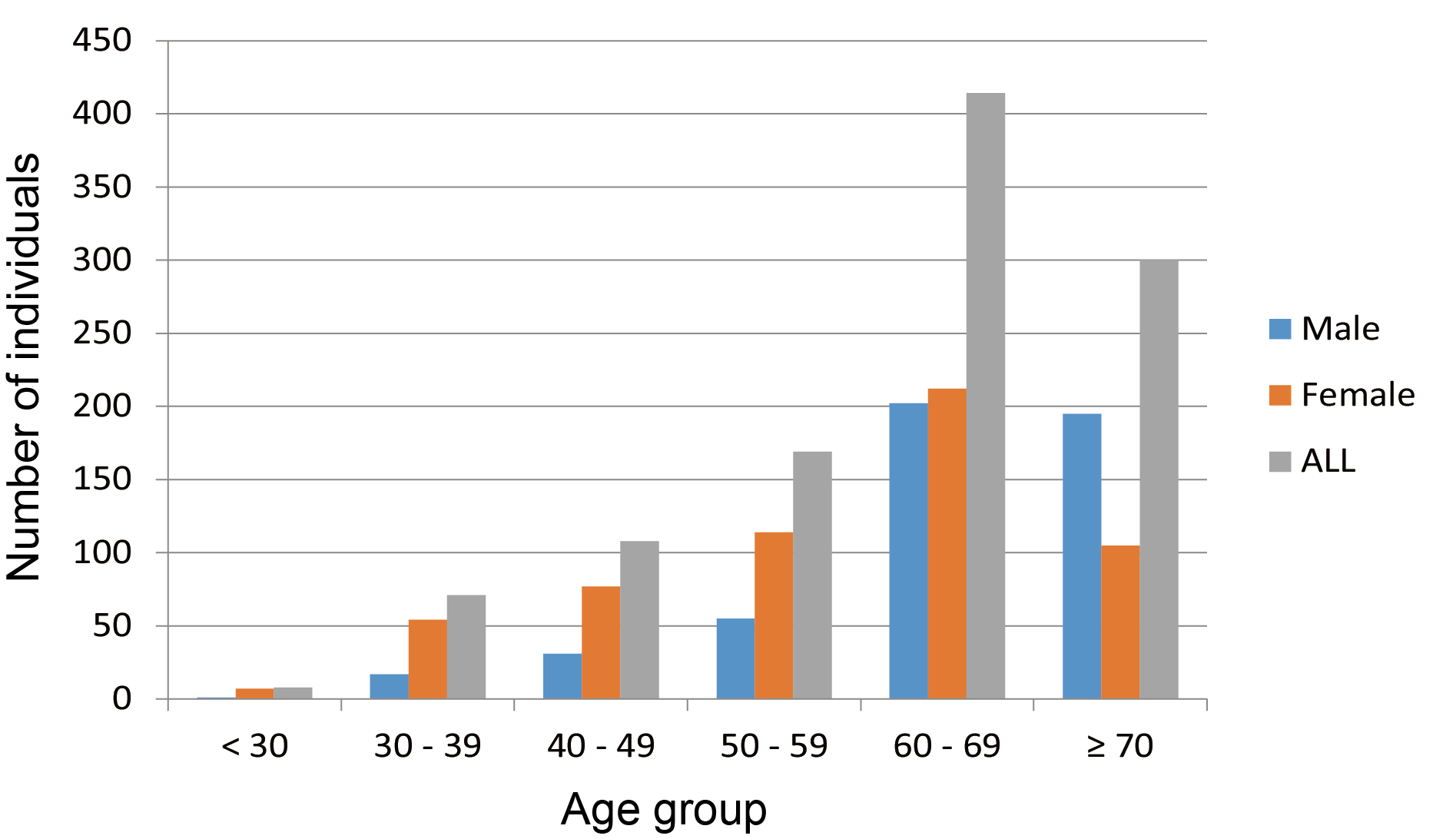

Supplement: Supplementary Figure 1 [file hgv201550-s1.doc]
