# Supplementary material for: iJGVD: an integrative Japanese genome variation database based on whole-genome sequencing
Source: Hum Genome Var. 2015 Nov 26;2:15050–. doi: 10.1038/hgv.2015.50 (PMC4785574; doi:10.1038/hgv.2015.50)

**Supplementary Figure 2**

QQ-plots of comparison of allele frequencies

Top: Allele frequency comparison between ToMMo 1KJPN and HapMap JPT

Bottom: Allele frequency comparison of HapMap JPT between SNP genotype data and NGS data (1KGP).


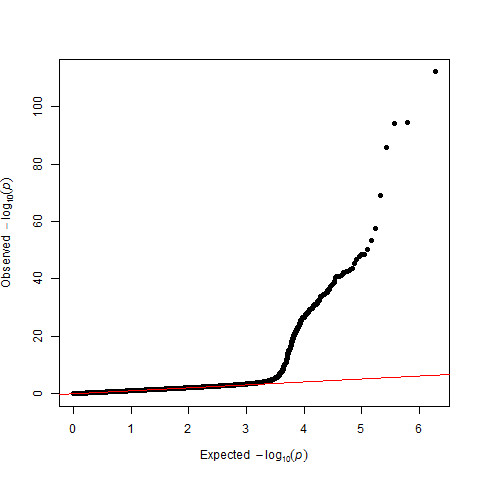

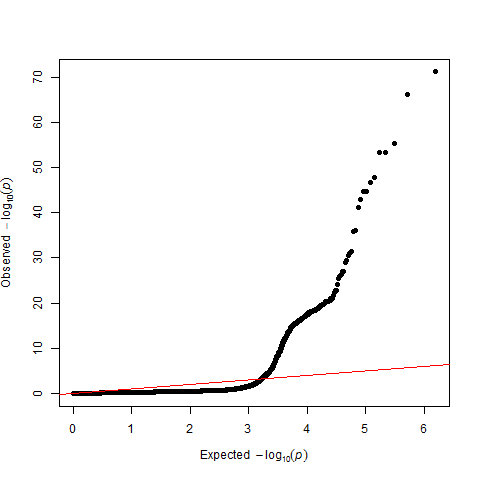

Supplement: Supplementary Figure 2 [file hgv201550-s2.doc]
